# Image memorability is linked to facilitated perceptual and semantic processing

**DOI:** 10.1162/imag_a_00281

**Published:** 2024-08-28

**Authors:** Will Deng, Diane M. Beck, Kara D. Federmeier

**Affiliations:** Department of Psychology, University of Illinois Urbana-Champaign, Champaign, IL, United States; Program in Neuroscience, University of Illinois Urbana-Champaign, Champaign, IL, United States; Beckman Institute for Advanced Science and Technology, University of Illinois Urbana-Champaign, Champaign, IL, United States

**Keywords:** image memorability, perception, semantic processing, event-related brain potentials, N300, N400

## Abstract

Strikingly, some images are consistently more likely to be remembered compared to others—a stable, intrinsic image property that has been termed image memorability. However, the properties that afford this memory advantage have remained elusive. In prior work, we showed that more memorable images are easier to perceive, and modeling work further suggests that semantic properties contribute to variance in memorability. Thus, we hypothesize that image memorability effects arise at the interface between perception and semantic memory. To test this hypothesis, we used event-related potentials (ERPs) to measure perceptual template matching (N300) and semantic access (N400) processes in a continuous recognition memory task using high and low memorability images, each repeated once. On initial presentation, both N300 and N400 amplitudes were less negative for high memorability images, showing that memorability is linked to both facilitated high-level perceptual processing and more efficient semantic activation. High memorability images also elicited a larger N300 repetition effect compared to low memorability images, revealing that their perceptual processing benefits more from the prior exposure. The results support the idea that images that better match visual templates and elicit more targeted semantic activations are easier to identify when encountered again, and further point to a potential interplay between semantic activation and perceptual matching in supporting image memorability.

## 1 Introduction

Image memorability is an item-level property that describes the likelihood that someone will recognize that image when seeing it a second time. It has often been estimated using a continuous recognition task wherein participants are presented with a sequence of new and repeated images with delayed repetition and asked to identify the repeats (Bainbridge et al., 2013;Bylinskii et al., 2015;Isola et al., 2014;Khosla et al., 2015); memorability is then measured as the average hit rate (or*d*-prime;Bainbridge, 2017;Bainbridge & Rissman, 2018) for each item’s repetition. Importantly, memorability scores for a given image have been found to be consistent across participants, such that iterated split-half analyses show high correlations—for example, a Spearman’s rank correlation of 0.75 for a diverse set of images (Isola et al., 2011) and 0.68 for a more constrained set of unfamiliar face images (Bainbridge et al., 2013). Additionally, convolutional neural network models trained on images and image labels have been able to produce reliable memorability estimates, which supports memorability as an intrinsic item property (Khosla et al., 2015;Needell & Bainbridge, 2022).

Despite the consistency in image memorability and the fact that models can accurately predict which images are more memorable, it has proven difficult to identify specific image properties that can explain the memorability effect. Both behavioral and fMRI experiments have shown that basic image features, such as color and spatial frequency, do not reliably predict memorability (Bainbridge et al., 2017;Bylinskii et al., 2015;Isola et al., 2014). Variation in memorability is also not strongly correlated with subjective judgments of aesthetics or interest (Isola et al., 2014) and has been shown to be distinct from top-down cognitive control such as depth of encoding and priming (Bainbridge, 2020;Wakeland-Hart et al., 2022). Neural network models that incorporate both perceptual (e.g., regions within the images) and semantic (e.g., image category) features can produce more accurate memorability estimates than models that only integrate perceptual features (Khosla et al., 2015;Needell & Bainbridge, 2022), suggesting that memorability effects may lie at the interface between high-level perceptual and semantic processing.

In our previous study (Deng et al., 2024), we investigated whether memorability may be related to statistical regularity—for example, the degree to which information in an image matches learned visual patterns. Many models of visual recognition propose that the visual system engages in perceptual hypothesis testing (Bar, 2003;Bullier, 2001;Hochstein & Ahissar, 2002;Rao & Ballard, 1999), in which incoming visual input is compared to an internal, memory-based prediction as to what that input might be. One such model proposes that these internal predictions reflect real-world statistical regularities, which are generalized representations that, on average, allow the visual system to quickly connect the visual input to semantics (Beck et al., in press;Center et al., 2022,^2024^;Kumar et al., 2021;Shao & Beck, 2024;Yang & Beck, 2023). On this view, statistically regular images should be easier to process, as they will more closely match the internal prediction and thus yield less prediction error. Indeed, previous work using an intact/scrambled task, wherein participants indicate whether a briefly presented stimulus is a regular image or noise, has shown that statistically regular stimuli are more readily perceived, yielding higher detection accuracy or requiring shorter presentation times to reach criterial levels of detection accuracy (Caddigan et al., 2017;Center et al., 2022;Greene et al., 2015;Smith & Loschky, 2019;Yang & Beck, 2023). For example, scene images that have been judged to be better examples of scene categories (e.g., forest, city street, beach) are more readily perceived than less representative scenes (Caddigan et al., 2017), and this has been linked to facilitated late-stage perceptual processing (Kumar et al., 2021). Given that, like statistically regular images (Shao & Beck, 2024;Torralbo et al., 2013), high memorability images have been found to elicit more similar neural activation patterns compared to low memorability images (Bainbridge & Rissman, 2018;Bainbridge et al., 2017), it is possible that memorability might be linked to statistical regularity and thus affects ease of perceptual processing. Using a staircasing procedure to control presentation duration in an intact/scrambled task with high and low memorability images, we showed, and then replicated with a second stimulus set, that high memorability images require shorter presentation time to be detected (Deng et al., 2024). Thus, memorability is associated with facilitated perceptual processing, possibly because more memorable images better match memory-based templates invoked during perception. A similar ease of processing argument has been put forward by others (Broers et al., 2018;Goetschalckx et al., 2019;Han et al., 2023).

Memorability, then, may arise from processing differences at the interface wherein high-level perceptual information comes to be linked into long-term memory. Indeed, as mentioned previously, neural networks models that include conceptual as well as perceptual features can predict image memorability more accurately compared to those that only include perceptual features (Khosla et al., 2015;Needell & Bainbridge, 2022), and recent work has suggested that semantic properties may actually contribute more than visual properties to memorability for objects (Kramer et al., 2023). Thus, the nature or organization of the semantic information evoked by images may play a critical role in allowing these images to later be recognized more accurately. Further understanding of image memorability effects, therefore, would benefit from the use of event-related potentials (ERPs), which can separately measure perceptual and semantic aspects of processing evoked by images. Here, we assess image memorability effects using ERPs, focusing in particular on two ERP components, the N300 and N400, that have been associated, respectively, with high-level visual processing and long-term semantic memory access.

Because high memorability images were more readily perceived in our previous work (Deng et al., 2024), we hypothesize that memorability may modulate the amplitude of the N300 component. The N300 is a negative-going ERP response with a broadly frontal distribution that peaks around 300 ms post-stimulus-onset. It is elicited by images such as line drawings and photographs of objects (Barrett & Rugg, 1990;McPherson & Holcomb, 1999), scenes (Kumar et al., 2021), and faces (Jemel et al., 1999). Its amplitude is reduced (less negative) for images that are easier to recognize and/or categorize. For example,Schendan and Kutas (2002)presented participants with fragmented line drawings of objects and gradually decreased the levels of fragmentation; they found that N300 amplitudes were smaller for images that were closer to being identified.

Based on findings like these, Schendan and colleagues have theorized that N300 amplitudes are linked to how readily the visual features of a stimulus can be matched to the long-term memory representation of known objects (Schendan, 2019;Schendan & Lucia, 2010). More recently, similar patterns of N300 effects have also been observed for scenes: Scenes that are good exemplars of their scene category—which, as described earlier, are detected more readily (Caddigan et al., 2017)—elicit smaller (less negative) N300s than do bad exemplars (Kumar et al., 2021). Thus,Kumar et al. (2021)suggested that the N300 might be a more general index of the ease and/or success with which incoming visual information can be matched to learned statistical regularities during perception. Given our finding that high memorability images behave like statistically regular images in a detection task, we expect that they might also elicit reduced N300s compared to low memorability images. If so, this would support the idea that more memorable images better match perceptual templates, making it easier to connect those images with long-term memory representations.

Using ERPs, we can also probe the relationship between memorability and semantic processing, as indexed by the N400, another negative-going component that peaks after the N300 (around 400 ms) and that has been linked to the access of information from long-term, multimodal (i.e., semantic) memory (Kutas & Federmeier, 2011). N400s are observed in all types of meaningful stimuli, including visual, auditory, and signed words (Kutas et al., 1987), environmental sounds (Van Petten & Rheinfelder, 1995), and line drawings, photographs, and movies (Ganis et al., 1996;Holcomb & McPherson, 1994;McPherson & Holcomb, 1999;Sitnikova et al., 2008). N400 amplitudes are reduced (become less negative) by a variety of factors that impact processing at the level of meaning. For example, it has been well established that N400 amplitudes show a graded sensitivity to the fit between an incoming stimulus and its context, with reduced (less negative) N400s to stimuli that are related to a prior “prime” stimulus or that are more expected in a sentence, discourse, picture series, or movie (e.g.,Hamm et al., 2002;Kutas & Hillyard, 1980; see review inKutas & Federmeier, 2011). This effect is thought to arise because N400 amplitudes reflect the amount of new semantic information brought on-line by the incoming stimulus, which is correspondingly reduced when some of that information has already been activated during the processing of the context (Federmeier, 2021).

This account also explains systematic differences in N400 amplitudes to stimuli out of context (e.g., in lists). For example, for words, N400 amplitudes are graded by orthographic neighborhood size, a measure of how similar an incoming string is to other known lexical items. Both meaningful (e.g., words and acronyms) and non-meaningful (e.g., pseudowords and illegal letter strings) strings of letters elicit larger (more negative) N400 amplitudes when they have higher numbers of orthographic neighbors (Laszlo & Federmeier, 2011). One explanation for this pattern is that, when an incoming letter string is being processed, it activates semantics across the network of similar (confusable) forms, such that stimuli from a denser neighborhood activate more net semantic information, reflected in more negative N400 amplitudes (Holcomb et al., 2002). The same idea can also apply to semantic activation during image processing, wherein unrecognizable objects (Supp et al., 2005) and improbable images (Proverbio & Riva, 2009) also elicit more negative N400 amplitudes. When an image cannot be clearly comprehended, it may not be efficiently mapped onto specific semantic information and thus will elicit a higher, and possibly more variable, set of semantic activations. Extending these ideas to memorability, it is notable that low memorability images elicit neural activation patterns that are less similar to one another than those of high memorability images, which may also indicate a more variable set of semantic activations for the less memorable images (Bainbridge & Rissman, 2018;Bainbridge et al., 2017). If high memorability images better map to the activation of a specific set of semantic features, which also makes them more recognizable when viewed again, then they may also elicit smaller N400 amplitudes than do low memorability images.

We thus hypothesized that memorability might affect the amplitude of both the N300 and N400 components, with more positive amplitudes on both components for high versus low memorability images, even on initial presentation, reflecting differences in how these images are processed at the junction between high-level perception and long-term memory access. However, the fact that memorability is generally assessed based on recognition rate when images are presented for a*second*time makes it important to also examine ERP responses to image repetitions. We therefore adopted a continuous recognition design, presenting images of varying memorability twice across the stimulus stream and asking participants to indicate for each image whether it is new or old.

Both the N300 (Eddy et al., 2006) and N400 (Kutas & Federmeier, 2011) components have been shown to yield repetition effects in the form of less negative amplitudes when stimuli are presented for a second time after a brief delay. The N300 repetition effect has been postulated to be related to activation of learned object representations (Schendan & Maher, 2009). Canonical views of objects, which can be identified faster and more accurately because they better match stored object representations, elicit larger repetition effects (Schendan & Kutas, 2003); the same objects in non-canonical views have been found to have reduced or even absent repetition effects (Eddy & Holcomb, 2011). In turn, N400 repetition effects have been linked to the implicit maintenance of semantic/conceptual information from prior exposure (Olichney et al., 2000;Voss & Paller, 2007). Thus, we expect to see basic repetition effects on both components for all images. Of interest, then, is whether memorability might interact with the size of the repetition effect. Given that recognition rates are higher for high memorability images, we might expect to also see enhanced repetition effects for these items, suggesting that the boost to recognition at the behavioral level may be associated with processing fluency in high-level perceptual processing (N300), semantic processing (N400), or both.

Although our hypotheses are focused on the N300 and N400, we will also measure effect patterns on the late positive component (LPC), as this component has been extensively studied in the context of memory (Addante et al., 2012;Bermúdez-Margaretto et al., 2015;Curran, 2000;Finnigan et al., 2002;Voss & Paller, 2007). The LPC is a positive-going component with a posterior distribution that peaks around 500-800 ms (after the N400). It has been associated with explicit recollection (Curran, 2000;Finnigan et al., 2002) and recognition confidence (Addante et al., 2012). Thus, we expect that high, compared to low, memorability images would elicit more positive LPC amplitudes on repetition and possibly even on first presentation, given work on subsequent memory effects, which finds that more positive responses on initial presentation are predictive of later memory success (reviewed inMecklinger & Kamp, 2023).

## 2 Methods

### 2.1 Participants

We determined the current study’s sample size based on the effect size estimate from a previous study that found a statistical-regularity-based N300 effect for visual scenes (Kumar et al., 2021); since N300 effects tend to be smaller than N400 effects, powering the study based on the N300 should also ensure sufficient power for N400 effects. An*apriori*power analysis using G*Power 3.1.9.7 for a paired sample t-test indicated that we needed 24 participants to detect an effect size of*d*= .6 with 80% power and a significance criterion of*α*= .05. To obtain the target sample size of 24, we recruited 25 participants from the University of Illinois, who were paid for their time. Written informed consent was obtained in accordance with procedures and protocols approved by the University of Illinois Institutional Review Board. Participants all self-reported to be right-handed, have normal or corrected-to-normal vision, and no history of head trauma, seizures, neurological, or reading disorders. Data from one participant were excluded due to poor data quality, leaving 24 participants (16 self-identifying as female and 8 as male,*M_age_*= 26) for analysis.

### 2.2 Materials

One hundred and fifty high memorability and 150 low memorability images were selected from the LaMem dataset using the rankings provided by the researchers (Khosla et al., 2015; seeFig. 1for image examples); these were identical to the images used in Experiment 1 ofDeng et al. (2024). The same number of images for each of five experimenter-determined categories (animal, architecture, nature, object, and people) were selected for the two conditions to broadly control for any category effect (Bylinskii et al., 2015). Images were resized to 512 x 512 pixels, and the contrast was balanced between high and low memorability conditions. The memorability difference between the post-processed high and low memorability images was verified using the ResMem model with an estimated hit rate of 0.89 (range: 0.64–0.98,*SD*= 0.06) for the high memorability images and 0.53 (range: 0.41–0.75,*SD*= 0.09) for the low memorability images (Needell & Bainbridge, 2022). Thirty filler images (needed to create the lag structure) were selected from the same dataset with the same number of images in each of the five categories and resized to 512 x 512 pixels. The images were arranged so that every target image (high and low memorability images) repeated once with a lag between 6 and 15 images, and the filler images never repeated.

**Fig. 1.**
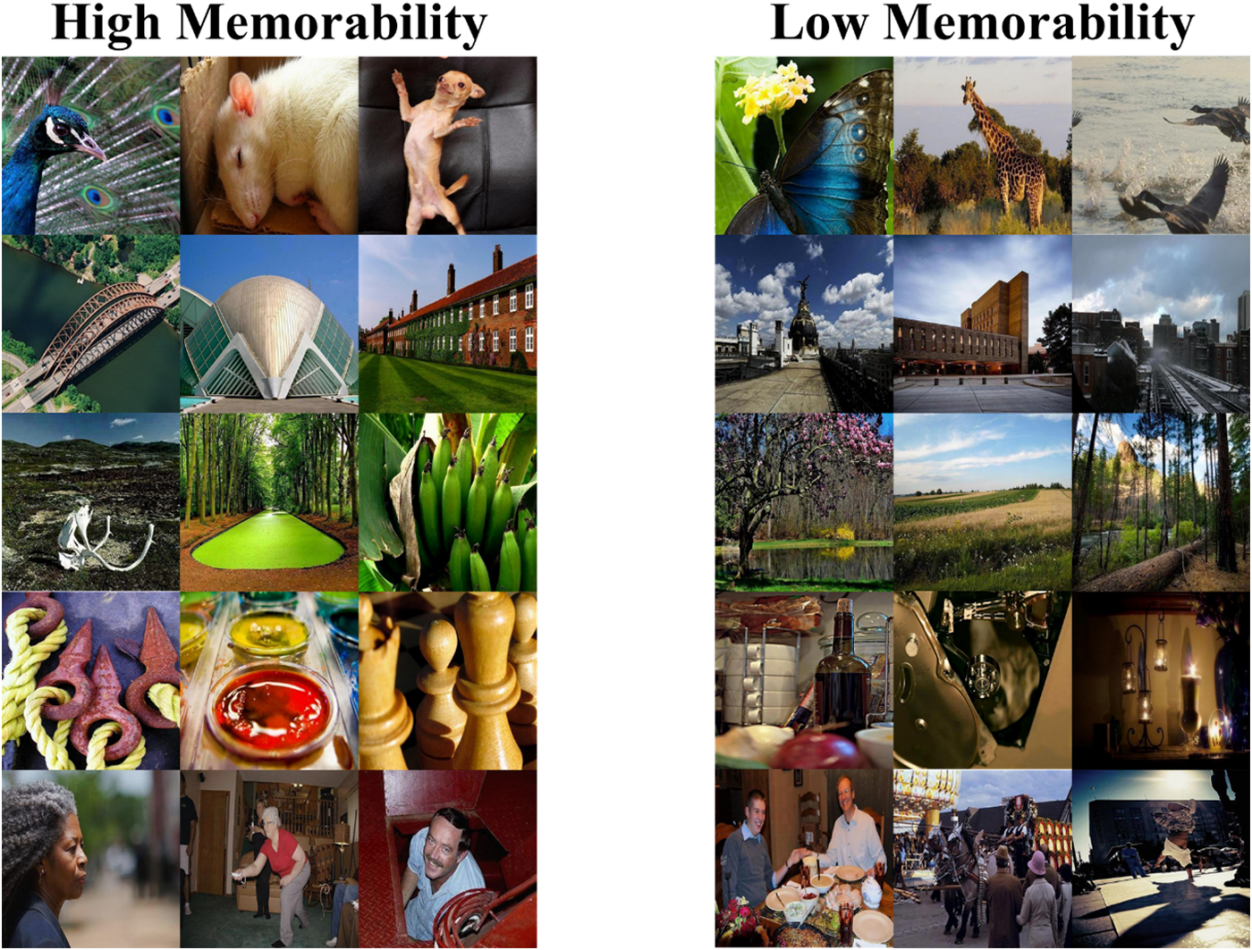
Examples of high and low memorability images from the LaMem dataset.

### 2.3 Procedures

At the beginning of the experiment, participants were informed that they would see a series of images one by one, some of which would repeat. They were then instructed to press a button with their right index finger for each new image and to press a different button with their right middle finger for each repeated image on a Cedrus RB-830 Response Pad (Cedrus, USA). The stimuli were presented on a 60 Hz CRT monitor of resolution 1280 x 1024 using the PsychoPy 2022.2.4 package (Peirce et al., 2019) and Python (Python Software Foundation. Python Language Reference, version 3.6.6). Participants completed the experiment sitting in a sound-attenuated recording booth, 118 cm away from the monitor. They were asked to remain still and to minimize eye movements and blinks during stimulus presentation. In each trial, a blue fixation cross at the center of the screen was displayed for 1000 ms against a black background, followed by an image for 1000 ms, and then another white fixation cross for 2000 ms. Participants were allowed to respond as soon as an image appeared on the screen and until a blue cross showed up for the next trial. Participants completed a 20-trial practice before the main experiment, which consisted of 630 trials divided into three blocks with the block order counterbalanced across participants.

### 2.4 EEG recording parameters

We obtained continuous EEG recordings using 26 silver/silver-chloride electrodes spaced evenly over the head and mounted in an elastic cap, amplified through a BrainAmpDC amplifier (Brain Products, USA). The 26 electrodes were Midline Prefrontal (MiPf), Left and Right Medial Prefrontal (LMPf and RMPf), Left and Right Lateral Prefrontal (LLPf and RLPf), Left and Right Medial Frontal (LMFr and RMFr), Left and Right Mediolateral Frontal (LDFr and RDFr), Left and Right Lateral Frontal (LLFr and RLFr), Midline Central (MiCe), Left and Right Medial Central (LMCe and RMCe), Left and Right Mediolateral Central (LDCe and RDCe), Midline Parietal (MiPa), Left and Right Mediolateral Parietal (LDPa and RDPa), Left and Right Lateral Temporal (LLTe and RLTe), Midline Occipital (MiOc), Left and Right Medial Occipital (LMOc and RMOc), and Left and Right Lateral Occipital (LLOc and RLOc); seeFigure 2. Additional electrodes placed on the outer canthus and infraorbital ridge of each eye were used to record blinks and saccades. Recordings were referenced on-line to the left mastoid and re-referenced off-line to the average of the left and right mastoids. Electrode impedances were reduced to below 5 kΩ. EEG was amplified and digitized with a 0.02–250 Hz band pass and a sampling rate of 1000 Hz.

**Fig. 2.**
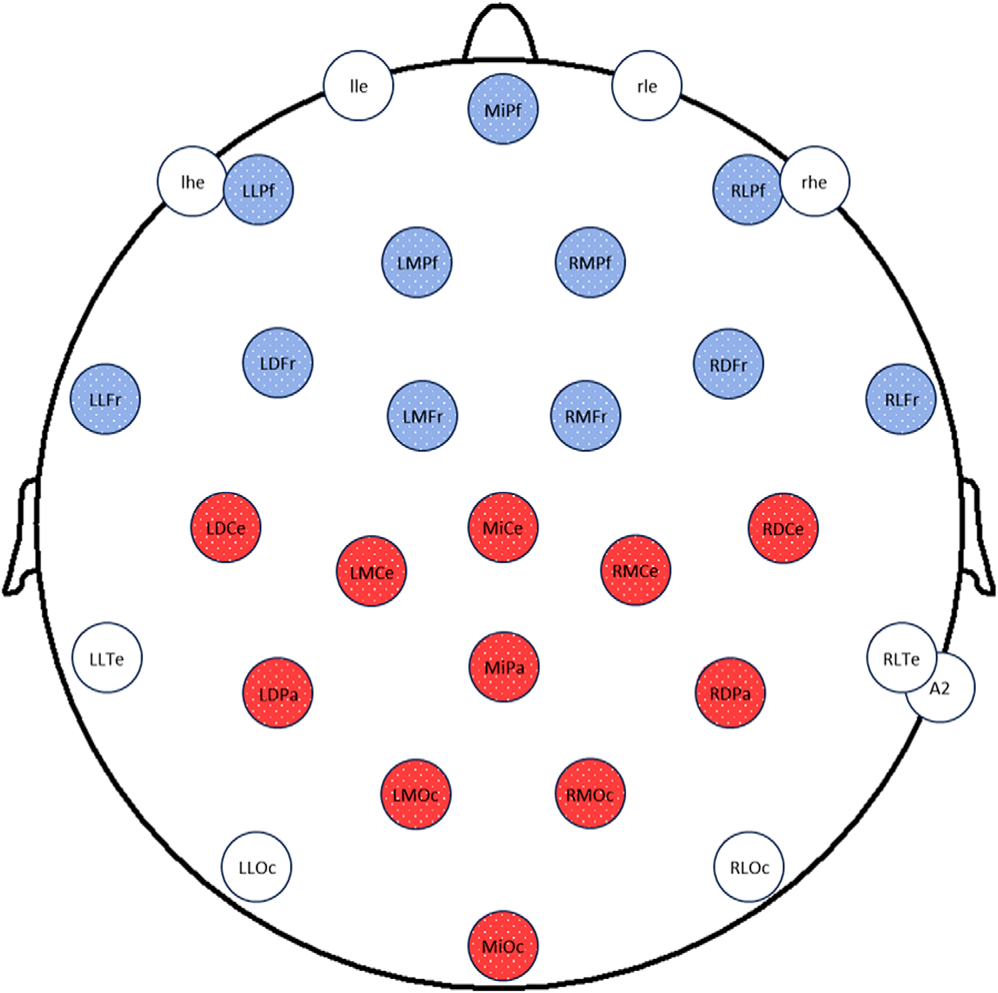
Electrode layout with frontal and central-posterior ROIs.*Note*. Blue circles indicate channels in the frontal ROI, and red circles indicate channels in the central-posterior ROI.

### 2.5 ERP data processing and analysis

Trial-level EEG data were processed with EEGLAB (Delorme & Makeig, 2004) and ERPLAB (Lopez-Calderon & Luck, 2014) toolboxes in Matlab. Each trial was a 1100 ms epoch from -200 to 900 ms time-locked to stimulus onset and baseline corrected with the mean amplitude of the 200 ms window prior to stimulus onset. We applied a 30 Hz low-pass filter and did artifact rejection for blinks, saccades, drift, and excessive muscle activity. Blinks and saccades were identified using thresholds calibrated for each participant in a condition-blind manner using visual inspection. Independent component analysis using AMICA was applied to participant data if there were blinks in more than 25% of the epochs (*N*= 6;Delorme et al., 2012); in this case, components were removed if they correlated with the eye channels at above 0.5. Epochs with artifacts that were not corrected were removed from all participant EEG data. Participants with greater than 25% of epochs removed due to artifacts even after applying ICA were excluded from analysis (*N*= 1 out of 25). On average, 85% of trials were retained with a range of 75–98% across participants (new high memorability:*N_trial_*= 130, Range = 114–149; old high memorability:*N_trial_*= 132, Range = 108–147; new low memorability:*N_trial_*= 122, Range = 92–147; old low memorability:*N_trial_*= 126, Range = 105–148).

To examine N300 effects, which are known to peak around 300 ms and to be largest over the front of the head (Hamm et al., 2002;Kumar et al., 2021;Schendan & Lucia, 2010), we computed mean amplitudes for each trial in an*apriori*250-350 ms time window over all 11 frontal channels (MiPF, LLPf, RLPf, LMPf, RMPf, LLFr, LDFr, LMFr, RMFr, RDFr, RLFr; seeFig. 2). N400 effects to color pictures also show a frontally skewed distribution (Federmeier & Kutas, 2002;Ganis et al., 1996), so we used this same frontal region of interest (ROI) to characterize the N400, measuring mean amplitudes in an*apriori*350–500 ms window. Given that the distribution of the N400 tends to be broad, for completeness, we also measured mean amplitudes between 350–500 ms over a central-posterior ROI (LDCe, LMCe, MiCe, RMCe, RDCe, LDPa, MiPa, RDPa, LMOc, MiOc, RMOc; seeFig. 2), with the expectation that this would show the same pattern as in the frontal ROI. We then used the same 11 channel posterior ROI to characterize the LPC, in an*apriori*time window of 500–800 ms. The single trial ERP signals were fitted to linear mixed-effect models with dummy-coded categorical fixed effects of old/new (repeated vs. new images) and memorability (high vs. low memorability), an interaction between old/new and memorability, random intercepts for participants and images, and random slopes of old/new and memorability for participants (Bates et al., 2014). Significance testing of fixed effects was estimated with t-tests using the Satterthwaite method in the lmerTest package in R (Kuznetsova et al., 2017). Because the ERP effects of primary interest precede decision making (and may be dissociable from behavioral patterns), we report analyses using all trials, not just correct trials. However, we confirmed that the results are unchanged if only correct trials are included.

## 3 Results

### 3.1 Behavioral results

To check for behavioral effects of image memorability, we compared the hit rates between high and low memorability images with a non-parametric Wilcoxon signed-rank test (functionally similar to a Mann-Whitney U test;Field et al., 2012) due to a violation of normality. We also computed false alarm rates (FA) and*d’*. The average hit rate and*d’*of high memorability images (*M_Hit_*= .94,*M_FA_*= .04,*M_d’_*= 3.72) was higher than that for low memorability images (*M_Hit_*= .89,*M_FA_*= .09,*M_d’_*= 2.76), whereas false alarms rates were lower for high memorability images. We compared hit rates with a paired one-sided Wilcoxon signed-rank test:*M_diff_*= .05, 95% CI = [.03, .07],*V*= 283,*p*< .001,*r*= .78. Similar results were obtained when comparing*d’*:*M_diff_*= .96, 95% CI = [.73, 1.13],*V*= 300,*p*< .001,*r*= .88. Thus, as predicted, participants were more likely to recognize high memorability than low memorability images, even with the short repetition interval used in this study.

### 3.2 ERP results

Grand average ERPs grouped by memorability and old/new status at all 26 scalp electrode sites are shown inFigure 3.

**Fig. 3.**
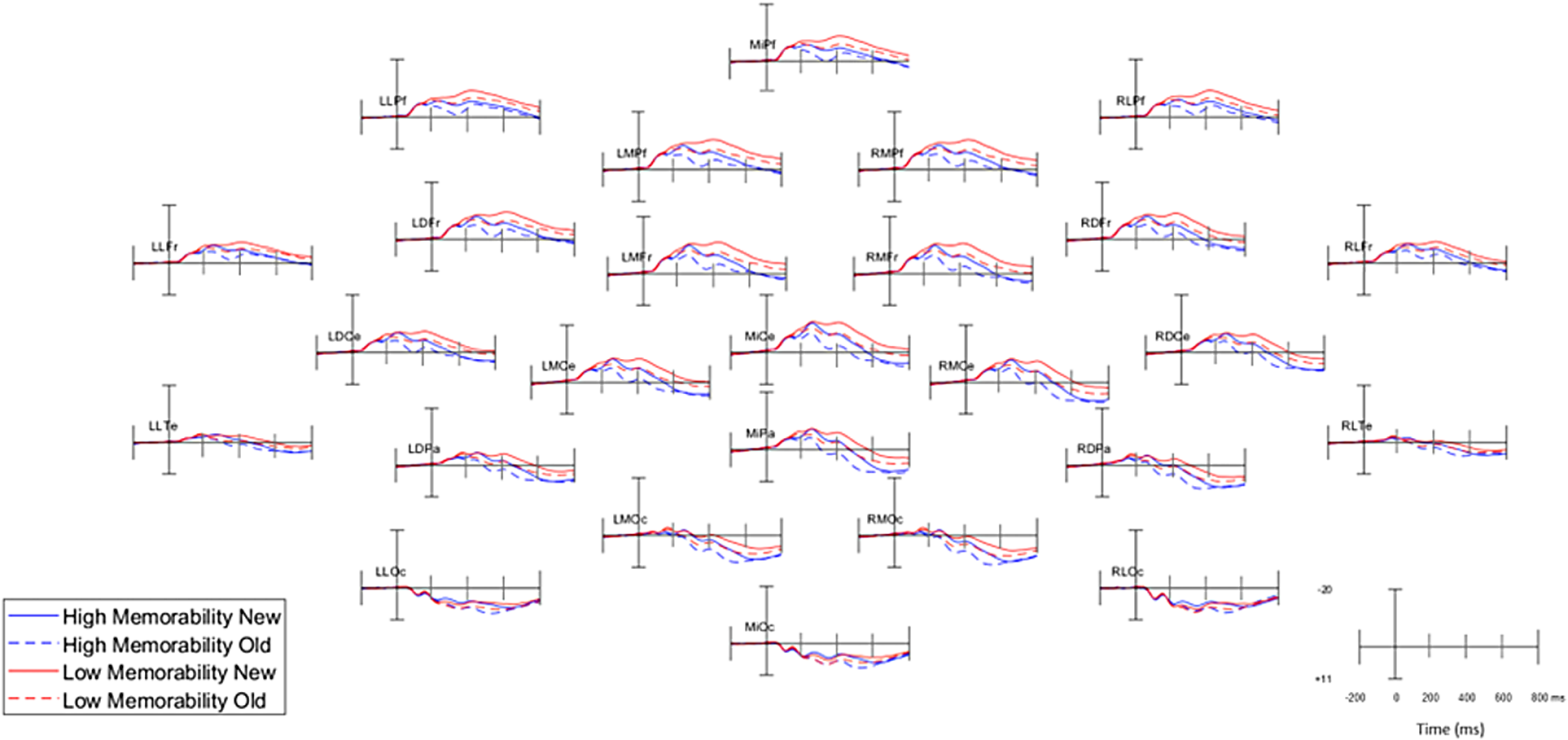
Grand average ERPs at all 26 electrode sites.*Note*. Plotted are responses to high memorability images (blue) and low memorability images (red) on initial presentation (New; solid lines) and when repeated (Old; dashed lines).

#### 3.2.1 N300

A mixed-effect model for the N300 time window with memorability and old/new as fixed effects revealed main effects of both memorability (*t*= 5.75,*p*< .001) and old/new status (*t*= 12.33,*p*< .001) and a significant interaction between the two (*t*= 3.53,*p*< .001). A simple comparison showed a reliable effect of memorability on the N300 even for first presentation of the pictures, such that new low memorability images (*M*= -8.25 µV) elicited more negative N300 amplitudes than new high memorability images (*M*= -6.11 µV),*M_diff_*= 2.14 µV,*t*= 5.75,*p*< .001. Repetition reduced N300 amplitudes overall, but the decrease in N300 amplitude from new to repeated items was 3.51 µV,*t*= 12.33,*p*< .001 for high memorability images and 2.54 µV,*t*= 8.79,*p*< .001 for low memorability images. The larger decrease in amplitudes from repetition for high memorability images (seeFigs. 4and5) resulted in an overall larger memorability effect on second presentation (second presentation N300 amplitudes of -5.71 µV for low memorability vs. -2.60 µV for high memorability).

**Fig. 4.**
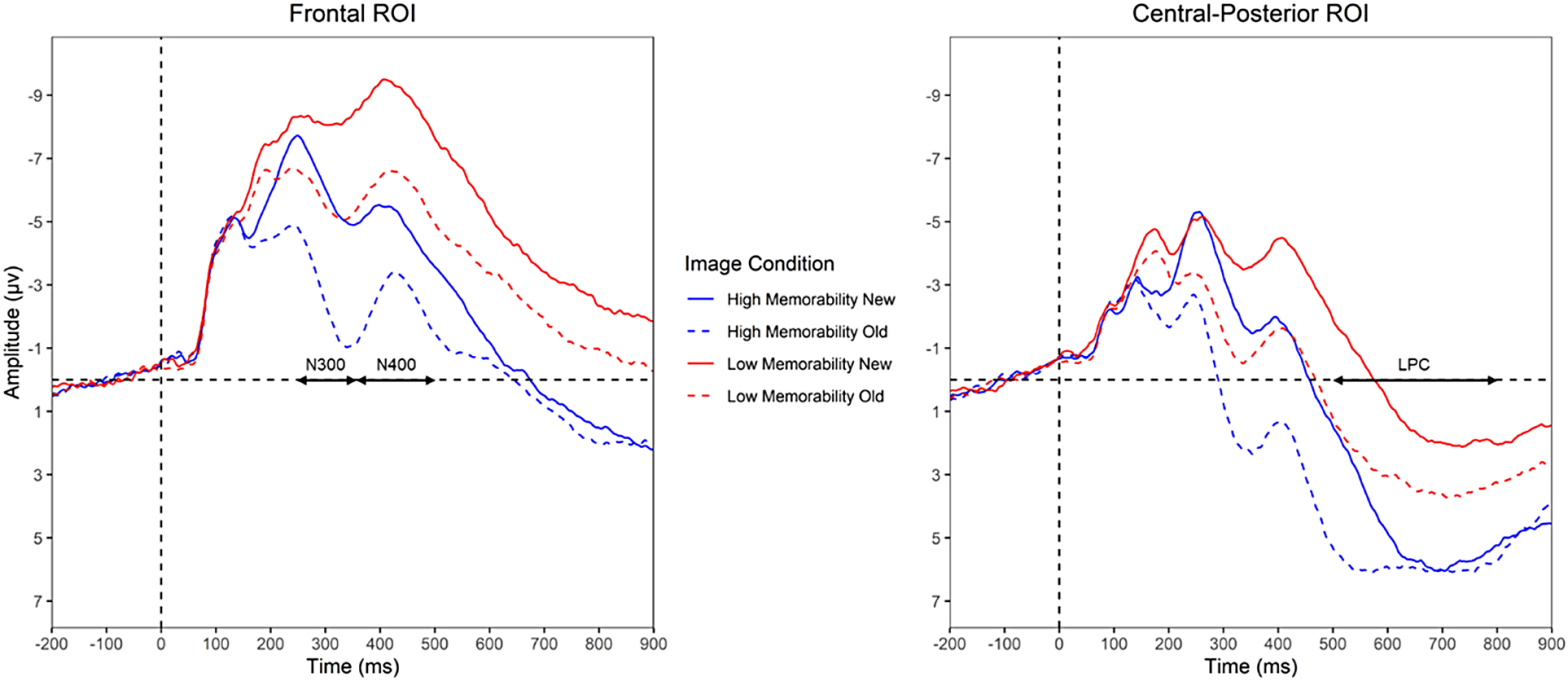
Grand average ERPs at the frontal and central-posterior ROIs.*Note*. Plotted are responses to high memorability images (blue) and low memorability images (red) on initial presentation (New; solid lines) and when repeated (Old; dashed lines). Time windows used to assess the N300, N400, and LPC are marked.

**Fig. 5.**
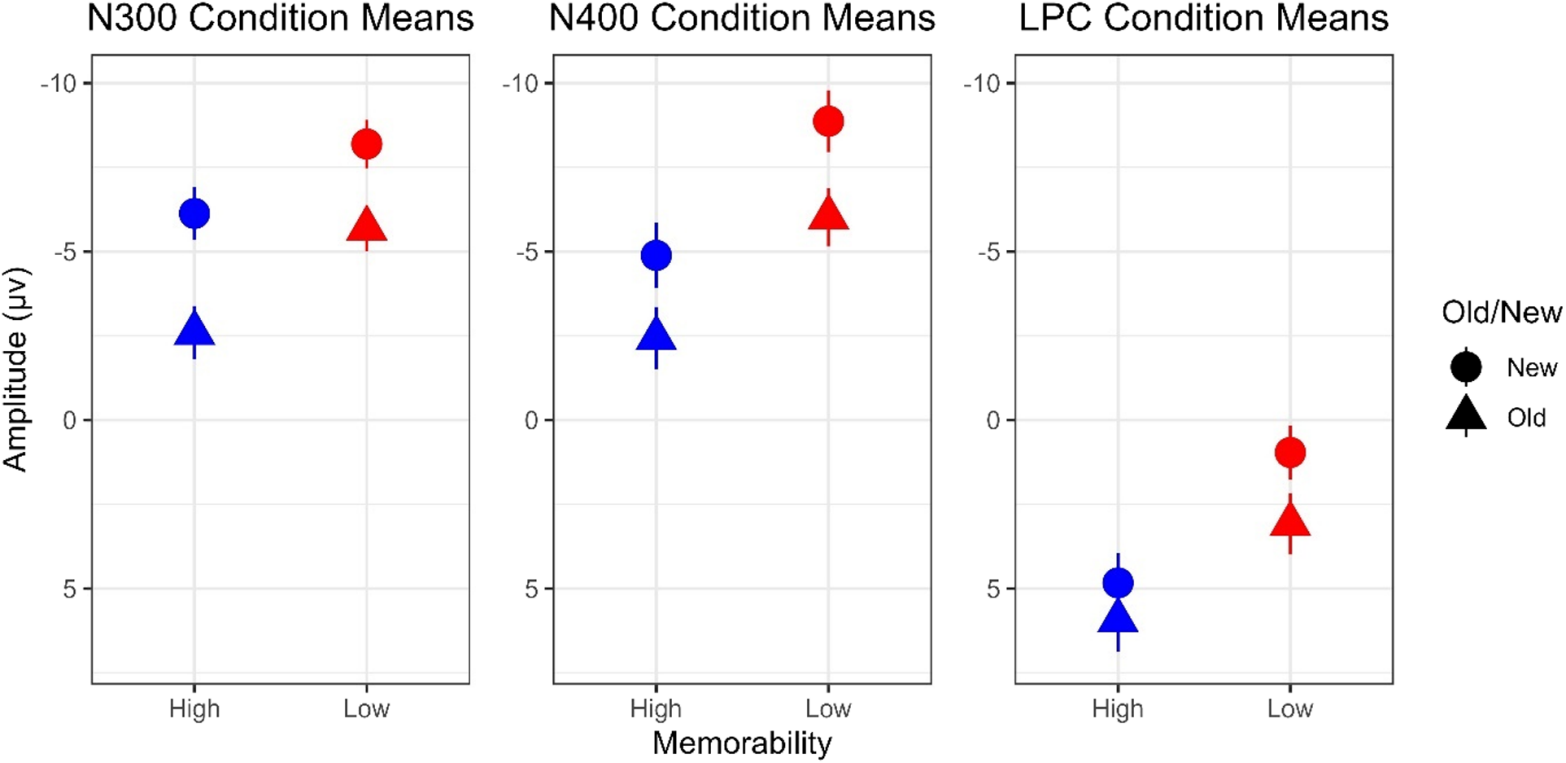
Average amplitudes of ROIs grouped by memorability and old/new status for the N300, N400, and LPC time windows.

#### 3.2.2 N400

The same mixed-effect model for the N400 time window at the frontal ROI revealed main effects of memorability (*t*= 11.96,*p*< .001) and old/new status (*t*= 8.33,*p*< .001) but no significant interaction (*t*= 1.61,*p*= .109). The memorability effect reflected the fact that low memorability images (*M*= -8.82 µV) elicited more negative N400 amplitudes than did high memorability images (*M*= -4.81 µV) on first presentation. N400 amplitudes were then decreased with repetition, to a similar degree for high and low memorability images (*M_diff_*= 2.63 µV). As predicted, the same N400 patterns were found in the posterior ROI—main effects of memorability (*t*= 9.57,*p*< .001) and old/new status (*t*= 8.77,*p*< .001), with no significant interaction (*t*= 1.71,*p*= .087).

#### 3.2.3 Relationship between N300 and N400

The differences in repetition effect patterns as a function of memorability for the N300 versus N400 attest that ERP patterns measured in the N300 and N400 windows do not reflect a single mechanism. However, given the spatial and temporal overlap between the two components, we conducted exploratory analyses to further separate them and understand their relationship. First, we confirmed that, even on first presentation, memorability still predicts N400 amplitudes even after the N300 is added as a covariate in the mixed-effects linear model (*t*= 10.15,*p*< .001); that is, the N400 differences cannot be explained away by the N300 differences.

We next examined whether N400 amplitudes on first presentation would predict the magnitude of the N300 repetition effect, which would suggest that the semantic activation on the first presentation impacts the subsequent repetition effects. To test it, we built a mixed-effects linear model with the N300 and N400 amplitudes on first presentation as fixed effects—the N300 amplitudes serve to control for the initial N300 differences on first presentation. Results suggest that the N400 amplitudes on first presentation predict the magnitude of the N300 repetition effect (*t*= 2.642,*p*= .008). Images that elicit smaller (less negative) N400 on first presentation produce larger repetition effect on second presentation.

#### 3.2.4 Lpc

The same mixed-effect model conducted in the LPC time window revealed main effects of memorability (*t*= 12.31,*p*< .001) and old/new status (*t*= 2.97,*p*= .005) and a significant interaction between the two (*t*= 3.76,*p*< .001). For new items, there was a memorability effect, with more positive responses to high (*M*= 4.84 µV) than to low (*M*= .91 µV) memorability images,*M_diff_*= 3.99 µV,*t*= 12.31,*p*< .001. Repetition led to more positive LPC amplitudes overall, but the increase in LPC amplitudes from new to repeated items was 1.08 µV,*t*= 2.97,*p*< .005 for high memorability images and 2.17 µV,*t*= 5.92,*p*< .001 for low memorability images. Thus, repetition effects on the LPC were enhanced for low compared to high memorability images. However, overall LPC amplitudes remained more positive for high compared to low memorability images on second presentation,*M_diff_*= 2.90,*t*= 8.99,*p*< .001.

## 4 Discussion

We used event-related potentials (ERPs) to investigate how high and low memorability images are processed at perceptual and semantic levels, both on first apprehension and when encountered for a second time. Previous findings suggest that high memorability images may also be more statistically regular, as, similar to patterns observed for other images characterized by higher statistical regularity—for example, objects seen from typical viewpoints and representative examples of natural scenes (Caddigan et al., 2017;Center et al., 2022;Greene et al., 2015;Smith & Loschky, 2019;Yang & Beck, 2023)—high memorability images are more readily perceived, requiring less time to be detected in an intact/scrambled task (Deng et al., 2024). Because statistical regularity is based on matching visual input to internal predictions from learned visual patterns (Beck et al., in press;Center et al., 2022,^2024^;Kumar et al., 2021;Rao & Ballard, 1999;Shao & Beck, 2024;Yang & Beck, 2023), we hypothesized that the facilitated perceptual processing for memorable images could be related to the activation of semantic information in long-term memory. Thus, our main analyses in the present work focused on the N300 and N400 ERP components, which have been associated with high-level perceptual processing (e.g., template matching;Center et al., 2024;Kumar et al., 2021) and semantic activation (Kutas & Federmeier, 2011), respectively. Because memorability measures the likelihood of recognizing an image that has been seen before, we used a continuous recognition task wherein a mixture of high and low memorability images was presented twice with a lag of 6-15 images, and participants were asked to indicate whether each image was new or repeated. Based on the ERP results for both the first and second presentations, we show that image memorability may arise due to an interplay between perceptual matching and semantic activation.

We predicted that the enhanced perceptual processing associated with memorability would be reflected in N300 amplitudes. Consistent with our prediction, at the first presentation, there was a small but significant N300 difference between the high and low memorability images. High memorability images elicited smaller (less negative) N300 amplitudes than did low memorability images, consistent with enhanced template matching that can aid in identification/recognition (Center et al., 2024;Kumar et al., 2021;Schendan & Kutas, 2002). The N300 difference coincides with our previous behavioral findings that high memorability images are more readily perceived (Deng et al., 2024). Perceptual processing differences could arise if there are differences in basic visual features across the high and low memorability images. Here, we controlled for size and color contrast and also matched the image sets at a broad categorical level but did not attempt to control for other possible low-level differences. However, prior studies have consistently found that memorability effects are not well predicted by lower-level image features (Bainbridge et al., 2017;Bylinskii et al., 2015;Isola et al., 2014). Thus, we interpret the N300 difference as more likely arising because high memorability images are more statistically regular. Previous work has shown that scenes that are more statistically regular are both more readily perceived (Caddigan et al., 2017) and elicit smaller N300s (Center et al., 2024;Kumar et al., 2021), identical to the pattern seen here for high (vs. low) memorability images. The commonality with statistical regularity is an interesting one. We have argued that real-world statistical regularities, that is, those regularities built up over a lifetime, should produce generalized benefit such that, on average, the visual system is able to more quickly connect the visual input to semantics (Beck et al., in press;Center et al., 2022,^2024^;Kumar et al., 2021;Shao & Beck, 2024;Yang & Beck, 2023). By extension, this experience-based perceptual fluency may also confer a memory advantage.

Moreover, the perceptual fluency associated with memorable images seems to be accompanied by differences in semantic processing. We were interested in possible memorability-based differences in semantic activation since semantic features of images have been found to improve model prediction of memorability scores (Khosla et al., 2015;Needell & Bainbridge, 2022) and may even contribute more to memorability than visual features (Kramer et al., 2023). Based on fMRI data showing more homogeneous neural activation patterns for high memorability images (Bainbridge & Rissman, 2018;Bainbridge et al., 2017) as well as smaller N400s for statistically regular images (Center et al., 2024;Kumar et al., 2021), we predicted that high memorability images may be more easily recognized because they are more efficiently mapped to specific semantic features. The more efficient and/or less variable mapping onto semantic information should thus result in an overall lower level of semantic activation, which would affect N400 amplitudes (Kutas & Federmeier, 2011). Consistent with our prediction, we observed that high memorability images elicit significantly smaller (less negative) N400s at first presentation. This is similar to findings linking (frontally distributed) N400 responses to conceptual fluency, which has also been linked to better recollection (e.g.,Hou et al., 2013;Voss et al., 2010;Wang et al., 2015). Our finding here is important because, to our knowledge, this is the first direct evidence that image memorability is related to semantic activation patterns during initial processing.

Because memorability is based on population-level recognition performance from tasks wherein people need to identify an image when seeing it a second time, we also examined repetition effects on the N300 and N400. Repetition effects were measured as changes in amplitude from the first to the second presentation, which allows us to probe for changes during perceptual processing and semantic activation as a result of the previous exposure. Replicating past work, both the N300 and N400 components showed a basic repetition effect, with less negative amplitudes on second presentation, showing that both aspects of processing are facilitated by the prior exposure. The size of the N400 repetition effects did not vary based on memorability. However, there was a larger N300 repetition effect for high memorability images compared to low memorability images. Thus, the perceptual processing of high memorability images benefited more from repetition, consistent with earlier work showing that canonical views of objects elicit larger repetition effects (Schendan & Kutas, 2003). These results suggest that more easily remembered and more statistically regular images both benefit more from repetition, and that this benefit seemed to be localized at the level of late-stage perceptual processing indexed by the N300 (cf.Schendan & Maher, 2009).

High memorability images, therefore, are characterized by a reduced level of semantic activation at both the first and second presentation and by having particularly enhanced perceptual processing after initial exposure. One possibility is that the patterns of semantic and perceptual processing are linked, in that more targeted semantic activation may help to refine/restrict the perceptual templates to which the visual input is matched, which, in turn, facilitates perceptual processing at the second presentation. In contrast, less focused and more extensive semantic activation may be less informative feedback to perceptual templates. This is consistent with studies showing that, although knowledge of learned image categories can lead to faster visual perception (Maier & Rahman, 2019), additional semantic information, such as the category’s functional significance, can actually hamper instead of facilitate perception, leading to slower processing times (Maier et al., 2014). Thus, a large amount of semantic information activated by an image may in fact create a perceptual disadvantage. In turn, precision at the perceptual-semantic interface can affect memory. For example, two-tone face images designed so that they appear like faces to all participants (well-defined semantically for the face category) are more likely to be recognized at the second presentation than images that were less well defined and thus may not be seen as faces by all participants (Brady et al., 2019).

It is possible, then, that semantic features make important contributions to memorability because semantic information serves the critical function of training the representations that determine how readily images are perceived/recognized later on. Our exploratory analyses provide some support for the idea that the initial semantic analysis of the images affects their perceptual processing on subsequent exposures. We found that the N400 amplitudes on first presentation predict the strength of the N300 repetition effect, specifically, images that elicit smaller (less negative) N400 on first presentation also undergo a larger reduction in N300 amplitudes in subsequent exposure. However, additional work is needed to replicate this pattern and to further probe whether perceptual and semantic processing affect memorability independently or interactively and, if the latter, what the precise nature of that interaction might be.

Besides the N300 and N400 components, which were the main focus of this study, we also examined the late positive component (LPC), which follows the N400 in time, because it has been associated with explicit recollection (Curran, 2000;Finnigan et al., 2002) and recognition confidence (Addante et al., 2012) in memory tasks, even for stimuli without learned semantics (Bermúdez-Margaretto et al., 2015;Voss & Paller, 2007). On the first presentation, high memorability images elicited larger (more positive) LPC signals, which likely reflects high encoding fluency of item-specific information that has been found to affect later recognition (Mecklinger & Kamp, 2023). Additionally, prior work using recognition paradigms has also found that LPC responses are graded not only by recent exposure, but also by cumulative lifetime exposure (Yang et al., 2019). Thus, this pattern provides further support for the idea that higher memorability images may tend to be more statistically regular. The LPC repetition effect differed between the memorability conditions: Responses to low memorability images showed a typical “old/new effect,” with increased LPC amplitudes on second presentation compared to first presentation, suggesting facilitation in memory associated with stimulus repetition. Different from the pattern for low memorability images, high memorability images did not show a repetition effect on the LPC. The smaller repetition effect in the high memorability condition is likely not a reflection of attenuated memory retrieval (as contradicted by behavioral results) but, instead, is due to the higher encoding fluency (more positive LPC) at the first presentation limiting the amount of further memory facilitation through repetition. Indeed, even though repetition had a larger impact on LPC responses for low than for high memorability images, LPC amplitudes on second presentation were still more positive for high memorability images, suggesting better recollection and/or higher memory confidence (consistent with behavioral patterns). Overall, LPC responses thus provide additional evidence supporting enhanced memory encoding and retrieval for high memorability images, possibly because they are more similar to past experience.

In conclusion, we not only confirmed that high memorability is associated with facilitated perceptual processing but also extended prior work on memorability by showing memorability-based differences in the amount of semantic activation. Critically, we found that high memorability images, which are associated with higher recognition rate, actually trigger less semantic activation. Coupled with the facilitated perceptual processing, a picture emerges whereby the perceptual features of more memorable images not only map more efficiently onto object representations but also the associated concepts themselves are more well defined and easier to access. This allows us to propose a potential interplay between semantic activation and perceptual processing, where more efficient semantic activation facilitates the formation and refinement of effective templates for perception. Such efficiency and precision results in more memorable items.

## Data Availability

Data and analysis code of this study can be accessed at the Open Science Framework athttps://osf.io/juy7a/?view_only=f9cf166c7d7b449faec7e0748ba6c088.
